# The Impact of Cold and Heat on Years of Life Lost in a Northwestern Chinese City with Temperate Continental Climate

**DOI:** 10.3390/ijerph16193529

**Published:** 2019-09-20

**Authors:** Jiangtao Liu, Yueling Ma, Yuhong Wang, Sheng Li, Shuyu Liu, Xiaotao He, Lanyu Li, Lei Guo, Jingping Niu, Bin Luo, Kai Zhang

**Affiliations:** 1 Institute of Occupational Health and Environmental Health, School of Public Health, Lanzhou University, Lanzhou 730000, Gansu, China; liujt2012@lzu.edu.cn (J.L.); ylma2017@lzu.edu.cn (Y.M.); xthe13@lzu.edu.cn (X.H.); lily2018@lzu.edu.cn (L.L.); guol16@lzu.edu.cn (L.G.); niujingp@lzu.edu.cn (J.N.); 2 Lanzhou Municipal Center for Disease Control, Lanzhou 730000, Gansu, China; wyhlzjk@126.com (Y.W.); lisheng76@sohu.com (S.L.); 3 Gansu Provincial Center for Disease Control and Prevention, Lanzhou 730000, Gansu, China; lsy1984120@163.com; 4 Shanghai Key Laboratory of Meteorology and Health, Shanghai Meteorological Service, Shanghai 200030, China; 5 Department of Epidemiology, Human Genetics and Environmental Sciences, School of Public Health, The University of Texas Health Science Center at Houston, Houston, TX 77030, USA; kai.zhang@uth.tmc.edu; 6 Southwest Center for Occupational and Environmental Health, School of Public Health, The University of Texas Health Science Center at Houston, Houston, TX 77030, USA

**Keywords:** years of life lost, distributed lag non-linear models, heat effects, cold effects, temperate continental climate

## Abstract

Cold spells and heat waves in a changing climate are well known as great public-health concerns due to their adverse effects on human health. However, very few studies have quantified health impacts of heat and cold in the region of Northwestern China. The purpose of the present study was to evaluate the effects of cold and heat on years of life lost (YLL) in Lanzhou, a city with temperate continental climate. We compiled a daily dataset including deaths, weather variables, and air pollutants in Lanzhou, China, from 2014–2017. We used a distributed lag non-linear model to estimate single-day and cumulative effects of heat and cold on daily YLL. Results indicated that both cold and heat were associated with increased YLL for registered residents in Lanzhou. Estimated heat effects appeared immediately in the first two days, while estimated cold effects lasted over a longer period (up to 30 days). Cold significantly increased the YLL of all residents except for males and those with respiratory diseases (≥65 years). Our results showed that both heat and cold had more pronounced effects on cardiovascular diseases compared to respiratory diseases. Males might be more vulnerable to heat, while females might suffer more YLL from cold. The effects of cold or heat on the elderly might appear earlier and last longer than those for other age groups.

## 1. Introduction

A changing climate leads to higher occurrences of extreme weather, including cold spells and heat waves, which are well known as great public-health concerns due to their adverse effects on health [1,2,3,4]. Many studies have examined the associations between temperature and cause-specific mortality. A study including data from 384 locations indicated that most of the temperature-related mortality burden was attributable to cold [5], which was consistent with findings from a national study in China [6]. Similar studies have extensively described the relationship between mortality and temperature as a U, V, or J shape. Mortality risk and burden are higher in specific subgroups, such as children and the elderly [6,7,8]. In addition, susceptibility to cold and heat can be influenced by many factors, such as age, gender, education, or economic level [9,10,11,12,13].

Most of the previous studies on the effects of weather variables focused on the number of daily deaths in the subtropical regions with warmer climates, but neglected the age and its composition ratio, which could be solved by properly applying years of life lost (YLL) [14,15,16]. Huang et al. first reported that both heat and cold increased daily YLL in Brisbane, Australia [16], which is a subtropical city with overall mean maximum and minimum temperatures of 26.3 °C and 14.7 °C, respectively [17]. Similarly, Yang et al. dealt with data from Guangzhou City, China, with mean maximum and minimum temperatures of 34.2 °C and 6.3 °C during 2003–2007 [18]. In the Hubei study, the temperature from 1st to 99th temperature percentiles ranged from −0.5–32.4 °C [12], similar to that of Ningbo, China [19]. Li et al. analyzed the association between temperature and YLL in Tianjin, a city that, despite being in northern China, had a temperate, semi-humid monsoon climate with 25th temperature percentile of 7.5 °C and 75th of 28.4 °C during 2006–2011 [9], much warmer than Lanzhou’s climate.

The territory of Lanzhou is complex and diverse, with an altitude of >1500 m and typical temperate continental climate. Lanzhou is cold in winter (average monthly low temperatures ranged from −6.6 °C to −1.6 °C during 2004–2017) but not too hot in summer (average monthly high temperatures ranged from 22.8–26.0 °C during 2004–2017; Table S1). Until now, no study has explored the YLL-temperature relationship in Northwestern China. The present study is the first to evaluate the effects of cold and heat on YLL using data in this region, adding to our knowledge from temperate continental climate regions.

## 2. Methods

### 2.1. Data Collection

Lanzhou, located at latitude 36°03′ N, 103°40′ E, is composed of five districts and three counties. It is an important industrial base and transportation hub of northwestern China, with a population of about 4 million. Lanzhou has a temperate continental climate, with an average annual temperature of 10.3 °C and an average total annual rainfall of 327 mm [20].

#### 2.1.1. Death Data and YLL Calculation

**Figure 1:**
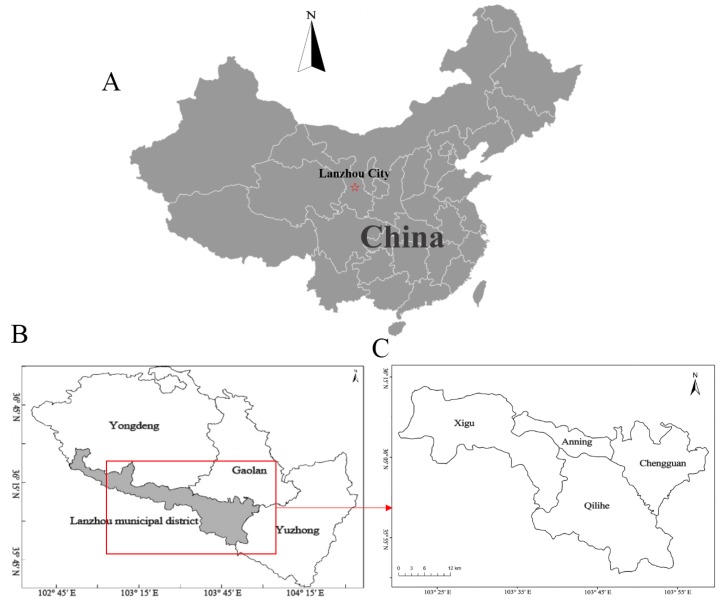
Geographical locations of Lanzhou City, China; (**A**,**B**) Locations of Lanzhou City, China; (**C**) The central four districts selected in the present study.

We obtained daily death data covering four central urban districts of Lanzhou City (Chengguan, Qilihe, Xigu, and Anning) during the period 1 January 2014 to 31 December 2017 from the Lanzhou municipal center for disease control (Figure 1). All deaths were the registered residents of Lanzhou City. The study was conducted in accordance with the declaration of Helsinki, and the protocol was approved by the ethics committee of Lanzhou University (Project identification code: IRB190210-1). Causes of death were classified into three subgroups according to the 10th Revision of the international classification of diseases (ICD-10), which were all non-accidental causes (ICD-10: A00-R99), cardiovascular (CV) diseases (ICD-10: I00-I99), and respiratory diseases (ICD10: J00-J99).

We obtained Chinese national life tables from the world health organization (WHO) for the years 2014–2016 [21]. YLL was calculated for each death by matching age and gender to the Chinese national life tables. We used life expectancy data from 2016 to calculate YLL for 2017, as data were unavailable for the latter year until now. Finally, we calculated daily YLL by summing YLL for all deaths on each day and stratifying them by gender, age, and specific cause of death. The calculation of YLL was done with the following formula:

YLL*_t_* = ∑ (deaths_*g*, *t*_ at age*_i_*) × (expected remaining life years at age*_i_* and year*_z_*)

where *t* is the day number in the study period (*t* = 1, 2, 3…, 1, 461); *g* is the gender; *i* is the death age; *z* is the year of death.

#### 2.1.2. Meteorological and Air Pollutant Data

The Lanzhou meteorological administration supplied meteorological data covering the four central urban districts, including daily maximum, mean, and minimum temperatures plus relative humidity. We obtained 24-h mean air pollutant data, including particulate matter with size ≤10 μm (PM_10_), nitrogen dioxide (NO_2_), carbon monoxide (CO), ozone (O_3_), and sulfur dioxide (SO_2_), from four air quality monitoring stations in Lanzhou. Both meteorological and air pollutant data were collected during 2014–2017.

### 2.2. Statistical Analysis

We performed a descriptive analysis to understand the time series characteristics of daily YLL, air pollutants, and meteorological variables over the study period.

The distributed lag non-linear models (DLNMs) used in our study represent a modeling framework for analyzing non-linear and delayed effects in time series data [22,23]. This framework is implemented in the R package “dlnm”, the functions of which construct a broad range of models within the DLNM family. Our initial analysis showed that daily YLL generally followed a normal distribution; therefore, the family function was Gaussian. Based on previous literature, we constructed our basic model according to this formula:

E(YLL*_t_*) = Cross-basis (temperature) + Cross-basis (pollutants) + NS (time, 7 × 4) + NS (humidity, 3) + DOW + Holiday + *α*,

where *t* is the day number in the study period (*t* = 1, 2, 3, …, 1, 461); YLL*_t_* is the sum YLL of observed daily death counts on day *t*; *α* is the intercept; NS (.) is a natural cubic spline; seven degrees of freedom (*dfs*) per year for time were used to control the long-term trend, and 3 *dfs* for relative humidity were used to control for potential confounding effects; DOW and Holiday are day of a week and official holidays in China, respectively.

We defined the cross-basis matrices for temperature and pollutants included in this study. The basis-functions we chose for the space of the factors were a cubic natural spline for the effects of pollutants; regarding the space of lags, we assumed a simple lag 0–1 parameterization. We included cross-basis matrices for all pollutants to one model. We specified a quadratic B spline in the cross-basis function of daily temperature. We placed knots for the spline at equally spaced values across the space of temperature, and the knots for lags at equally spaced values in the log scale of lags [24].

We estimated the single-day lag and cumulative effects of cold and heat and chose 30 days as the maximum lag [25,26].

In order to check the reliability of the above model and its results, we changed lag days for temperature (lag 15–30 days), *dfs* (6–10 per year) for time, and *dfs* (3–5) for relative humidity. We also assessed sensitivity to control for air pollutants.

Cold effects in the present study represented changes in YLL caused by the decreased temperature from the 25th to the 1st percentile of the overall temperature, while heat effects represented the changes of YLL caused by the increased temperature from the 75th to the 99th percentile of the overall temperature; this was consistent with other studies [25,27,28]. We performed all analyses with R software version 3.3.2 and used the “dlnm” package to conduct time series regression. Statistical significance was set at *p* value less than 0.05.

## 3. Results

### 3.1. General Results of Air Pollutants, Weather Variables, and YLL in Lanzhou City

According to the 2018 yearbook for Lanzhou, the average population during 2014–2017 was 3.23 million, and the average ratio (male/female) was 1.02, with the aging of Lanzhou’s residents [29]. Table 1 summarizes the daily statistics for air pollutants, weather variables, and YLL in Lanzhou from 2014–2017. A total of 42,942 deaths (32,032 of people ≥65 years) in Lanzhou were included. The mean daily YLL for the whole population was 420.5 years; males had more YLL than any other group (251.8), followed by people <65 years old who died due to non-accidental causes (224.8).

**Figure 2:**
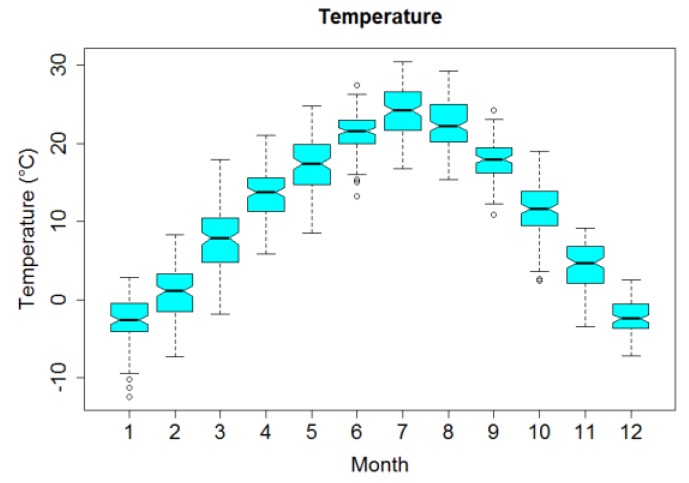
Box plots of monthly averages of temperature in Lanzhou, China, during 2014–2017.

Figure 2 displays box plots of monthly temperature. Mean temperature over the four years was 11.39 °C, ranging from −12.4 °C to 30.4 °C and showing an obvious seasonal trend.

**Figure 3:**
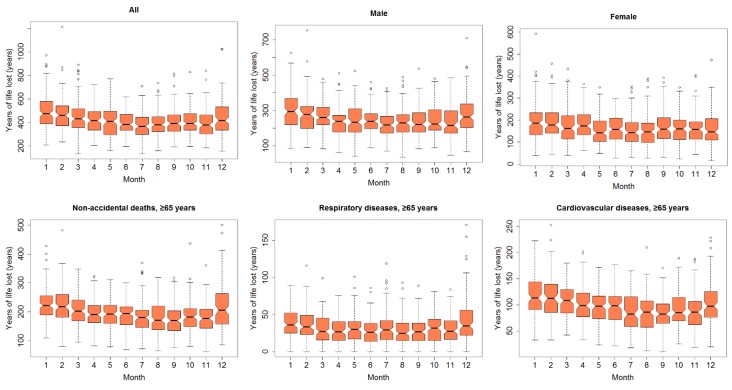
Box plots of monthly years of life lost (YLL) in Lanzhou, China, during 2014–2017.

Monthly YLL of various subgroups is shown in Figure 3 and Figure S1. As expected, YLL for these subgroups had a clear seasonal distribution, which was higher in colder months (December–March) and lower in warmer months (July–September).

### 3.2. Single-Day Lag Effects of Cold and Heat

**Figure 4:**
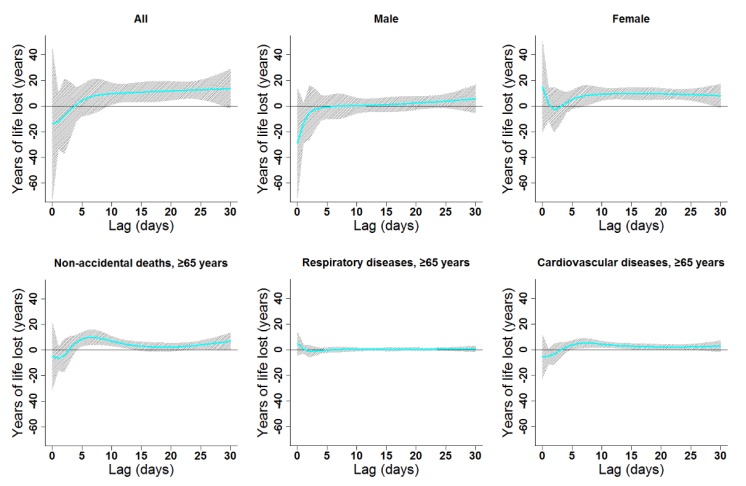
Lag patterns for single-day cold effects on various subgroups in Lanzhou, China. The bold lines represent the effect estimates, and the grey areas represent 95% confidence intervals. Cold effects in the present study represented changes in YLL caused by lower temperatures from the 25th to the 1st percentile of the overall temperature distribution. The reference temperature was 2.45 °C (25th percentile).

**Figure 5:**
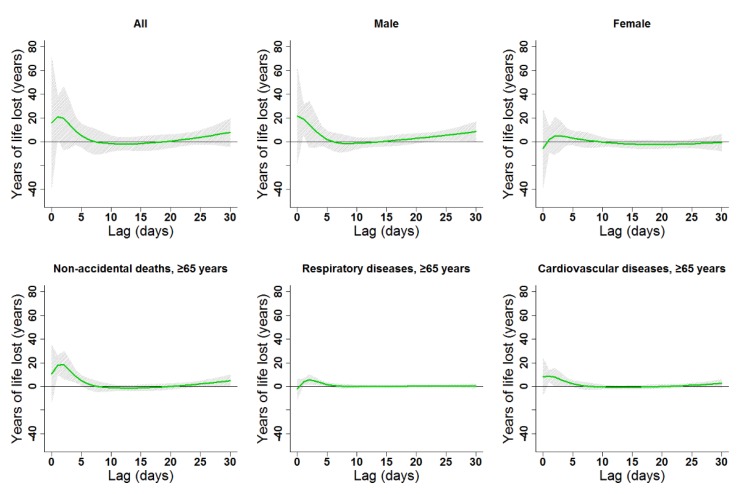
Lag patterns for single-day heat effects on various subgroups in Lanzhou, China. The bold lines represent the effect estimates, and the grey areas represent 95% confidence intervals. Heat effects in the present study represented changes in YLL caused by higher temperatures from the 75th to the 99th percentile of the overall temperature distribution. The reference temperature was 19.7 °C (75th percentile).

**Figure 6:**
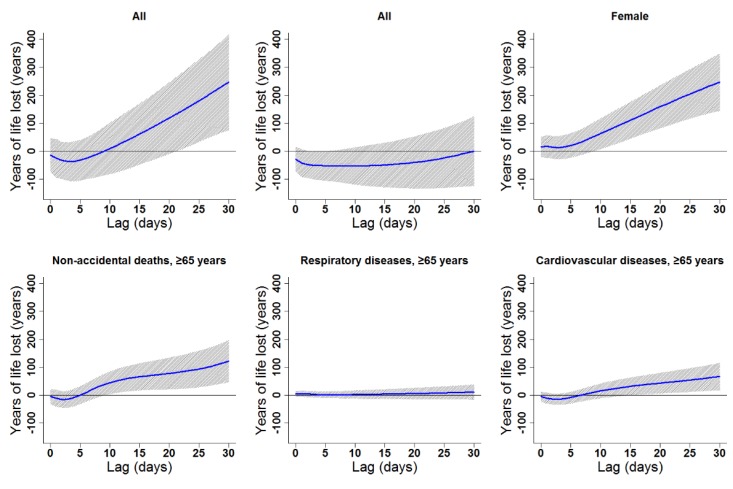
Lag patterns for cumulative cold effects on various subgroups in Lanzhou, China. The bold lines represent the effect estimates, and the grey areas represent 95% confidence intervals. Cold effects in the present study represented changes in YLL caused by lower temperatures from the 25th to the 1st percentile of the overall temperature distribution. The reference temperature was 2.45 °C (25th percentile).

**Figure 7:**
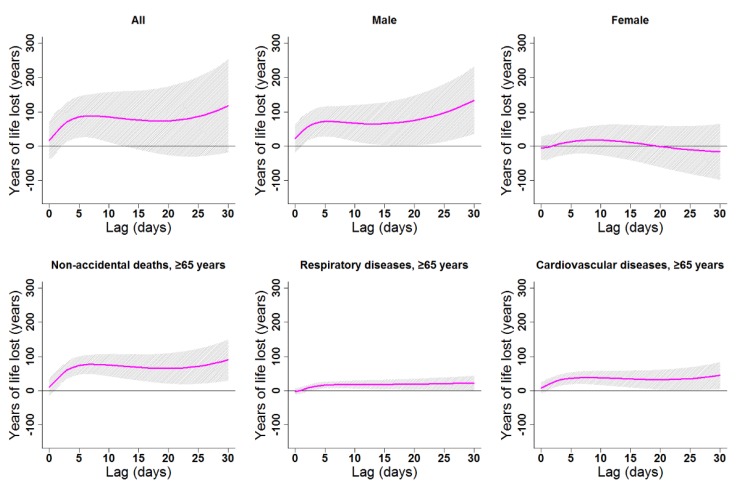
Lag patterns for cumulative heat effects on various subgroups in Lanzhou, China. The bold lines represent the effect estimates, and the grey areas represent 95% confidence intervals. Heat effects in the present study represented changes in YLL caused by higher temperatures from the 75th to the 99th percentile of the overall temperature range. The reference temperature was 19.7 °C (75th percentile).

The estimated effects of ambient temperature were non-linear for all subgroups and had various degrees of lag (Figure 4, Figure 5, Figure 6 and Figure 7, Figures S2–S5). In general, heat effects were strong and immediate, while cold effects were more delayed. The significant durations and the strongest single-day lag effects of cold and heat on YLL in the present study are shown in Table 2. Results indicated that males were more vulnerable to heat, while females suffered more from cold. Heat and cold effects lasted longer among people older than 65 years old compared to other age groups. Similar lag patterns were observed for different genders and diseases (Figure 4 and Figure 5, Figures S2 and S3).

### 3.3. The Cumulative Effects of Cold and Heat

Cumulative cold effects along lag 0–30 days were associated with increased YLL for all residents (246.87 years), females (246.41 years), the elderly in general (121.85 years), elderly women (76.83 years), and elderly with CV diseases (66.75 years; Table 3). The strongest effects of heat on the elderly (≥65 years) appeared in lags 0–30, 0–30, 0–8, 0–30, and 0–30 for non-accidental deaths, males, females, respiratory diseases, and CV diseases, respectively. YLL due to heat exposure were 89.82, 71.32, 30.11, 22.30, and 44.54 years for the elderly in general, elderly men, elderly women, elderly people with respiratory diseases, and elderly people with CV diseases, respectively (Table 3). Cumulative lag patterns were also slightly different for cold and heat (Figure 6 and Figure 7, Figures S4 and S5).

## 4. Discussion

Most studies dealing with the associations between YLL and temperature have focused on subtropical regions with warmer climates. It is important to conduct a study in those regions owing a typical temperate continental climate which is cold in winter but not too hot in summer. We applied a time series regression model combined with DLNM that allowed us to explore cumulative effects for a better view of the effects of ambient temperature, especially in relatively underdeveloped areas. Therefore, this study is unique and provides critical information to better understand the association between temperature and YLL in the temperate continental climate zone.

We found that both cold and heat increased YLL, indicating that the effects of both weather phenomena should receive attention even in a city with temperate continental climate. Single-day lag effects of cold on YLL in this study were as long as 5–29 days and the cumulative effect stabilized after some lags. Significant durations in our study seemed to be longer than in other studies conducted in regions such as Hong Kong or Taipei [31,32,33,34]. Lanzhou having colder weather, longer winters, and lower economic levels than other cities might have led to longer effects of cold on YLL in Lanzhou. In addition, we found that cold had stronger effects in the subgroups of females and the elderly, suggesting they had greater susceptibility to cold. Possible reasons might be that the elderly have relatively low immunity and many of them suffer from various underlying health problems, which make them more difficult to maintain homeostasis and adapt to environmental challenges [35]. The vulnerability of females to cold might be caused by differences from males in physiological functions, health status, thermoregulatory and adaptive capacity, and social status [11,12,36]. It might also be due to residual confounding of age, as there are greater numbers of women than of men within elderly age groups. In particular, these elderly women have reached menopause, which led them more likely to be affected by cold stress. A possible reason is decline in ovarian hormone concentrations during the menopausal transition and beyond, and increased risks of CV diseases [37,38].

For heat, the significant single-day lags were 1–5 days among various groups, as described above. The effects of heat seemed to be weaker than those of cold, reflecting Lanzhou’s above-mentioned climatic characteristics. Cumulative heat effects lasted longer for males and the elderly, indicating higher risks for these subgroups; this is in line with a study conducted in Tibet [32]. A possible explanation is that men are more likely to engage in outdoor jobs and activities, which increases their exposure to heat. Meanwhile, duration of heat effects was longer than in similar studies [10,11,28,39]. A previous study found that large communities with high population densities, low income per capita, and lack of nurses had a greater propensity toward increased risk of heat-related mortality displacement [40]. In addition, people in Lanzhou might be more sensitive to heat waves than those living in hotter areas due to their acclimatization to the temperate continental climate with cool summers and cold winters [41]. This indicates that more serious health issues will occur among Lanzhou residents as extreme weather events increase due to a changing climate. Consistent with the previous study [26], we found significant harvesting effects in heat. The harvesting effects might be modified by climates, socioeconomic, quality of medical service, and other factors [12,26]. Lanzhou is often recognized as a summer resort due to its cool weather from June to August with a mean temperature of 22.67 °C, so residents living in this cool climate in Lanzhou may be more vulnerable to short periods of hot days. This may lead to the significant harvesting effect of heat for death in Lanzhou. Our results also indicate the importance of the timely prevention or intervention actions to reduce heat related health effects.

As to YLL for specific diseases, the effects of cold and heat on the CV system are stronger than those on the respiratory system. This might have led to a higher YLL attributable to CV mortality in this study, consistent with other studies [42,43]. Exposure to cold increases numbers of erythrocytes and blood platelets and leads to an increase of blood cholesterol and fibrinogen [44,45], which contributes to CV diseases. Exposure to cold has also been found to increase the contraction of peripheral blood vessels, elevate blood pressure, increase the occurrence of coronary–artery spasm, and promote the occurrence of adverse CV events. Possible mechanisms linking heat with CVD include increased surface blood circulation and sweating, which lead to an increase in a series of physiological burdens [46].

Along with vulnerability in the elderly, higher frequency of cold or hot weather caused by climate change, projected population growth and an aging population is acknowledged to drive the rise in temperature-related deaths among the elderly [2], and these challenges also exist for China [6,47]. It is worth noting that elderly residents (≥65 years) in Lanzhou accounted for almost 10% in recent years, indicating a serious aging problem. Of people who died of non-accidental causes during 2014–2017 in Lanzhou, 74.6% were the elderly (≥65 years). What is worse, Lanzhou does not have a complete elderly-care system because of its poor socioeconomic status. Since the elderly of Lanzhou have already acclimated to its temperate climate, we assume that more and more of them would be affected by heat due to climate change. Therefore, more action should be taken to develop policy recommendations targeted at improving public health and protecting the elderly from changes in ambient temperature [48], particularly in a city like Lanzhou.

Lanzhou initiated a routine death surveillance system in 2011. In recent years, the data have become nearly complete, which allows us to conduct this study. Although many similar studies have been conducted in other regions, ours is the first to explore the relationship between YLL and ambient temperature in northwestern China. However, this study has some limitations to be considered. The data used were from only four central urban districts of Lanzhou, which might not be enough to represent all of northwestern China due to differences in geography. Therefore, studies using more city-level data and longer time periods might yield better estimates of the association between YLL and temperature in northwestern China. Our findings are further limited by a lack of control for other potential confounders, such as socioeconomic factors or lifestyles.

## 5. Conclusions

In summary, this study uniquely provides critical information to better understand the association between temperature and YLL in the temperate continental climate zone, especially in the Northwestern China. The results indicate that the cumulative effects of cold and heat on YLL are stronger than their single-day lag effects in Lanzhou. Males are more vulnerable to heat, while females suffer more YLL from cold. The effects of cold and heat on the elderly appear in earlier days and last longer than those for other age groups. Heat and cold exert stronger and more prominent effects on the CV system than those on the respiratory system. With an aging population, more attention should be paid to the elderly in Lanzhou, especially those with existing chronic diseases such as CV disease. Our findings suggest that prompt preventive measures or actions could help reduce heat-related health risks, while prolonged measures are needed to address cold-related health risks. Our findings can help improve clinical and public health practices to reduce the impact of possible extreme weather events.

## Figures and Tables

**Table 1:** Summary of statistics for air pollutants, weather variables, and YLL in Lanzhou, 2014–2017.

Variables	Daily Measures					
	Minimum	1st Quartile	Median	3rd Quartile	Maximum	Mean ± S.D.
PM_10_ (μg/m^3^)	19.72	81.73	107.30	145.86	1451.01	124.67 ± 86.07
NO_2_ (μg/m^3^)	12.25	37.70	50.39	63.74	143.12	52.97 ± 21.46
SO_2_ (μg/m^3^)	3.47	10.56	17.83	30.23	130.59	22.17 ± 15.19
CO (mg/m^3^)	0.34	0.81	1.08	1.61	4.59	1.33 ± 0.73
O_3_ (μg/m^3^)	0.79	30.79	46.56	63.35	125.05	48.43 ± 22.09
Temperature (°C)	−12.40	2.45	12.70	19.70	30.40	11.39 ± 9.4
Relative humidity (%)	16.00	39.00	50.00	61.00	94.00	51.51 ± 15.09
All	136.3	333.1	410.10	492.4	1211.0	420.5 ± 128.8
Male	35.5	188.9	242.6	303.6	753.3	251.8 ± 90.7
Female	15.5	114.8	160.2	210.1	593.0	168.8 ± 73.4
Non-accidental deaths (<65)	0	153.1	210.4	283.3	728.9	224.8 ± 104.4
Non-accidental deaths (≥65)	61.9	154.6	191.1	230.6	500.4	195.8 ± 58.6
Male (≥65)	6.6	77.4	101.8	127.3	283.4	104.2 ± 38.0
Female (≥65)	5.5	65.5	88.6	112.0	284.3	91.5 ± 36.0
Respiratory diseases (≥65)	0	17.4	29.2	43.4	170.7	32.2 ± 20.36
Cardiovascular diseases (≥65)	10.0	71.4	94.9	120.2	252.9	97.1 ± 36.3

Note: YLL: years of life lost; SD: standard deviation; PM_10_: particulate matter with size <10 μm; NO_2_: nitrogen dioxide; SO_2_: sulfur dioxide; CO: carbon monoxide; O_3_: ozone; 1st quartile: first quartile; 3rd quartile: third quartile; Guideline levels of WHO for each pollutant [30]: PM_10_ (24 h): 25 µg/m^3^; NO_2_ (1 h): 200 μg/m^3^; SO_2_ (24 h): 20 μg/m^3^; O_3_ (8 h): 100 μg/m^3^.

**Table 2:** Significant durations and strongest single-day lag effects of cold and heat for different subgroups in Lanzhou City.

	Significant Duration (Days)	Strongest Effects	Highest YLL (95% CI)
Cold effects (years)			
All	Lag 10–28	Lag 28	13.24 (1.53, 24.96)
Male	-	-	-
Female	Lag 8–29	Lag 16	9.76 (4.93, 14.59)
Non-accidental deaths (<65)	Lag 13–26	Lag 21	9.50 (3.58, 15.42)
Non-accidental deaths (≥65)	Lag 5–14, lag 23–29	Lag 7	10.01 (4.08, 15.94)
Male (≥65)	Lag 5–12	Lag 7	5.62 (1.59, 9.65)
Female (≥65)	Lag 5–14, lag 20–27	Lag 7	4.40 (0.52, 8.27)
Respiratory diseases (≥65)	-	-	-
Cardiovascular diseases (≥65)	Lag 5–25	Lag 7	5.14 (1.30, 8.97)
Heat effects (years)			
All	Lag 1	Lag 1	20.80 (3.03, 38.58)
Male	Lag 1, lag 22–30	Lag 1	19.06 (6.19, 31.94)
Female	-	-	-
Non-accidental deaths (<65)	-	-	-
Non-accidental deaths (≥65)	Lag 1 to 5	Lag 2	18.29 (6.51, 30.07)
Male (≥65)	Lag 1-5, lag 23–28	Lag 2	13.04 (5.03, 21.04)
Female (≥65)	Lag 1	Lag 1	6.44 (1.33, 11.55)
Respiratory diseases (≥65)	Lag 1–5	Lag 2	5.76 (1.59, 9.94)
Cardiovascular diseases (≥65)	Lag 1–4	Lag 1	8.72 (3.66, 13.78)

Note: “-” represents no statistical significance; CI: confidence interval.

**Table 3:** Significant durations and strongest cumulative lag effects of cold and heat for various subgroups in Lanzhou City.

	Significant Duration (Days)	Strongest Effects	Highest YLL (95% CI)
Cold effects (years)			
All	Lag 0–30	Lag 0–30	246.87 (75.09, 418.64)
Male	-	-	-
Female	Lag 0–9 to 30	Lag 0–30	246.41 (143.60, 349.22)
Non-accidental deaths (<65)	-	-	-
Non-accidental deaths (≥65)	Lag 0–10 to 30	Lag 0–30	121.85 (46.25, 197.44)
Male (≥65)	-	-	-
Female (≥65)	Lag 0–11 to 30	Lag 0–30	76.83 (27.43, 126.23)
Respiratory diseases (≥65)	-	-	-
Cardiovascular diseases (≥65)	Lag 0–15 to 30	Lag 0–30	66.75 (17.85, 115.66)
Heat effects (years)			
All	Lag 0–2 to 12	Lag 0–7	88.19 (25.43, 150.95)
Male	Lag 0–2 to 30	Lag 0–30	133.58 (35.01, 232.15)
Female	-	-	-
Non-accidental deaths (<65)	-	-	-
Non-accidental deaths (≥65)	Lag 0–1 to 30	Lag 0–30	89.82 (29.90, 149.74)
Male (≥65)	Lag 0–2 to 30	Lag 0–30	71.32 (30.61, 112.04)
Female (≥65)	Lag 0–2 to 14	Lag 0–8	30.11 (11.19, 49.03)
Respiratory diseases (≥65)	Lag 0–3 to 30	Lag 0–30	22.30 (1.09, 43.52)
Cardiovascular diseases (≥65)	Lag 0–2 to 30	Lag 0–30	44.54 (5.78, 82.30)

Note: “-” represents no statistical significance.

## Supplementary Materials

The following are available online at https://www.mdpi.com/1660-4601/16/19/3529/s1, Table S1: Monthly average temperature in Lanzhou City from 2004 to 2017 (°C); Figure S1: Box plots of monthly YLL (Non-accidental deaths (<65 years), Male (≥65 years), Female (≥65 years)) in Lanzhou City, China, during 2014–2017; Figure S2: Lag patterns for single-day cold effects on various subgroups (Non-accidental deaths (<65 years), Male (≥65 years), Female (≥65 years)) in Lanzhou City, China; Figure S3: Lag patterns for single-day heat effects on various subgroups (Non-accidental deaths (<65 years), Male (≥65 years), Female (≥65 years)) in Lanzhou City, China; Figure S4: Lag patterns for cumulative cold effects on various subgroups (Non-accidental deaths (<65 years), Male (≥65 years), Female (≥65 years)) in Lanzhou City, China; Figure S5: Lag patterns for cumulative heat effects on various subgroups (Non-accidental deaths (<65 years), Male (≥65 years), Female (≥65 years)) in Lanzhou City, China.
