# Supplementary material for: The Impact of Cold and Heat on Years of Life Lost in a Northwestern Chinese City with Temperate Continental Climate
Source: Int J Environ Res Public Health. 2019 Sep 20;16(19):3529. doi: 10.3390/ijerph16193529 (PMC6801473; doi:10.3390/ijerph16193529)
Supplement: Supplementary file 1 [file ijerph-16-03529-s001.pdf]

Supplementary material

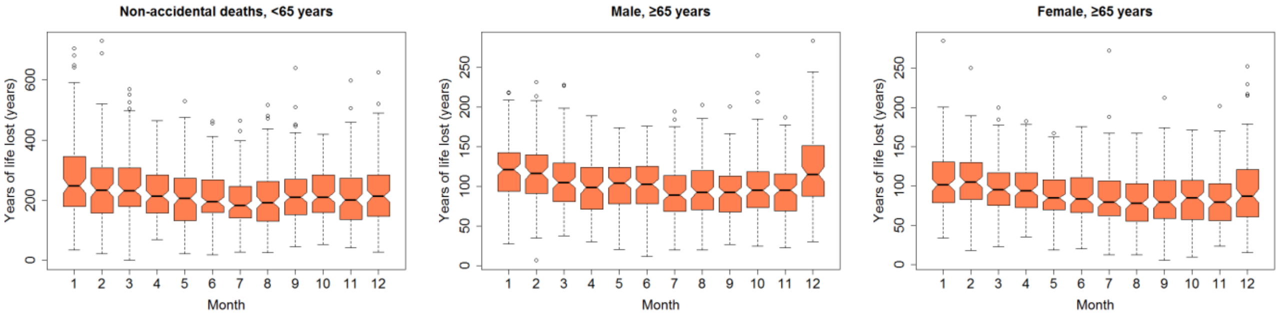

Figure S1. Box plots of monthly YLL (Non-accidental deaths (<65 years), Male (≥65 years), Female (≥65 years)) in Lanzhou City, China, during 2014–2017.

Table S1. Monthly average temperature in Lanzhou City from 2004 to 2017 (°C)[#]

Month	Year													
	2004	2005	2006	2007	2008	2009	2010	2011	2012	2013	2014	2015	2016	2017
Jan.	-3.5	-3.7	-3.0	-4.4	-6.6	-3.6	-2.8	-7.5	-5.9	-4.3	-2.8	-1.6	-4.4	-1.6
Feb.	1.3	-0.5	0.4	3.6	-4.1	3.2	0.9	1.4	-1.6	1.7	0.0	1.7	-0.5	2.2
Mar.	6.9	6.2	6.7	6.7	9.1	7.4	6.2	3.2	6.2	10.7	8.5	8.3	8.4	6.0
Apr.	14.9	13.9	13.1	12.2	13.8	15.1	11.1	14.4	14.0	14.2	12.8	13.7	14.4	13.1
May.	17.0	18.3	18.2	19.9	18.9	17.7	17.3	17.3	17.7	18.2	16.9	17.5	16.7	17.9
Jun.	21.1	22.9	22.7	21.2	21.9	23.5	21.0	22.4	22.8	22.0	21.5	21.5	21.9	21.1
Jul.	23.1	23.3	25.5	22.9	24.0	24.5	24.9	23.1	23.5	21.9	22.8	23.0	24.4	26.0
Aug.	22.2	22.4	24.8	22.9	20.9	20.9	23.1	23.2	23.1	23.4	20.5	23.1	25.1	21.2
Sept.	16.7	18.5	16.7	16.4	16.7	17.0	18.4	16.1	16.7	17.0	17.1	17.3	18.4	18.2
Oct.	10.6	9.9	13.3	9.4	11.4	11.3	10.5	11.2	11.0	12.4	11.8	12.2	11.5	10.5
Nov.	2.4	3.6	4.9	4.0	4.0	1.0	3.3	5.8	1.8	3.3	3.8	5.6	3.9	3.8
Dec.	-1.2	-4.8	-3.3	-1.2	-2.6	-3.0	-3.9	-3.1	-3.6	-3.4	-3.2	-2.2	-0.5	-2.6

Note: [#]the data were obtained from Lanzhou Meteorological Administration.

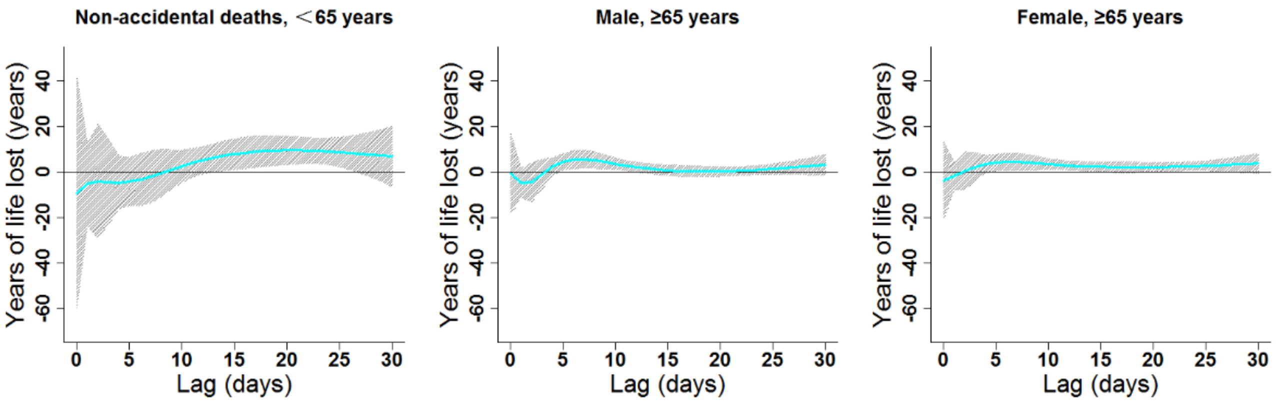

Figure S2. Lag patterns for single-day cold effects on various subgroups (Non-accidental deaths (<65 years), Male (≥65 years), Female (≥65 years)) in Lanzhou City, China. The bold lines represent the effect estimates, and the grey areas represent 95% confidence intervals. Cold effects in the present study represented changes in YLL caused by lower temperatures from the 25th to the 1st percentile of the overall temperature range. The reference temperature was 2.45°C (25th percentile).

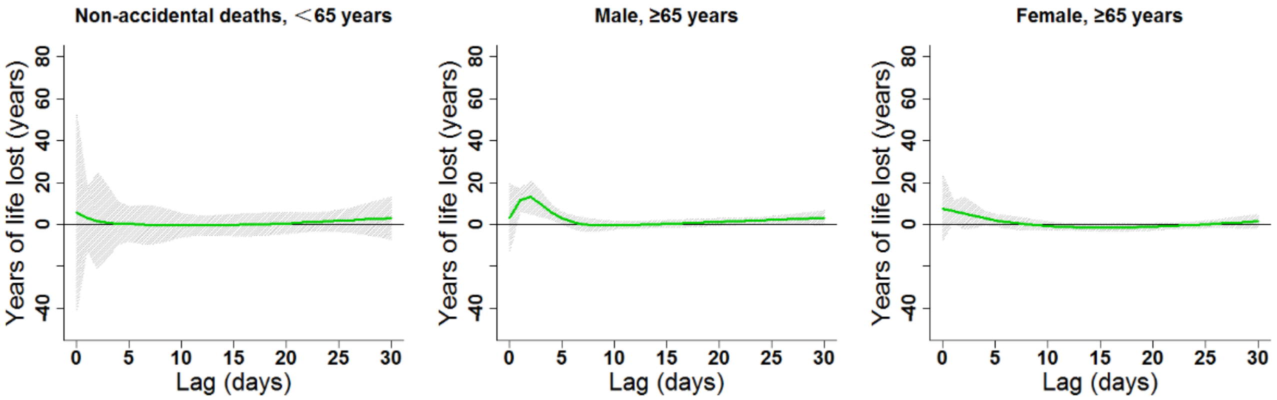

Figure S3. Lag patterns for single-day heat effects on various subgroups (Non-accidental deaths (<65 years), Male (≥65 years), Female (≥65 years)) in Lanzhou City, China. The bold lines represent the effect estimates, and the grey areas represent 95% confidence intervals. Heat effects in the present study represented changes in YLL caused by higher temperatures from the 75th to the 99th percentile of the overall temperature range. The reference temperature was 19.7° C (75th percentile).

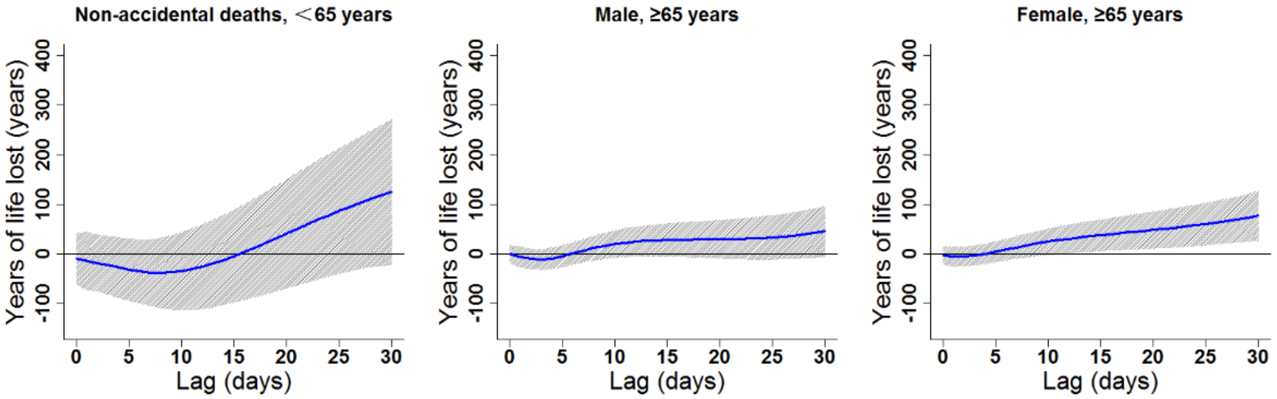

Figure S4. Lag patterns for cumulative cold effects on various subgroups (Non-accidental deaths (<65 years), Male (≥65 years), Female (≥65 years)) in Lanzhou City, China. The bold lines represent the effect estimates, and the grey areas represent 95% confidence intervals. Cold effects in the present study represented changes in YLL caused by lower temperatures from the 25th to the 1st percentile of the overall temperature range. The reference temperature was 2.45°C (25th percentile).

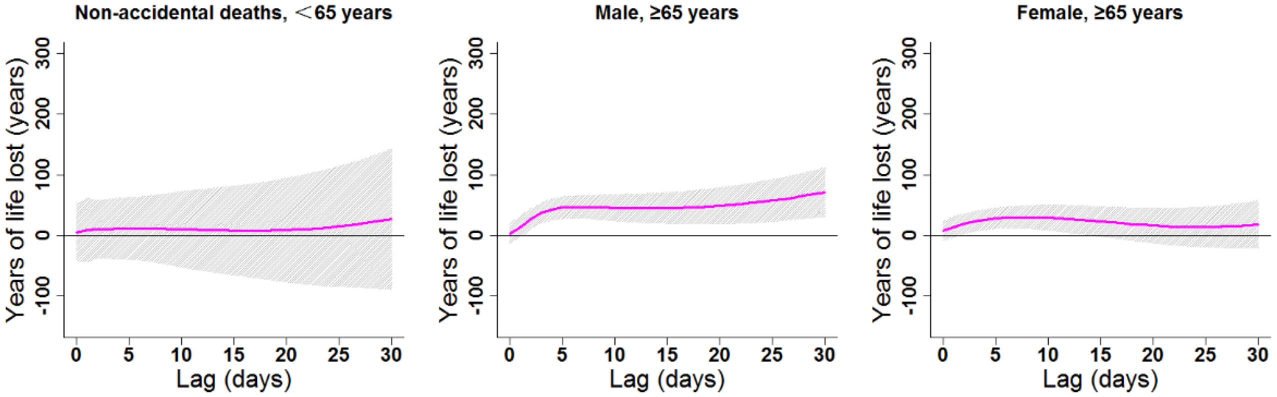

Figure S5. Lag patterns for cumulative heat effects on various subgroups (Non-accidental deaths (<65 years), Male (≥65 years), Female (≥65 years)) in Lanzhou City, China. The bold lines represent the effect estimates, and the grey areas represent 95% confidence intervals. Heat effects in the present study represented changes in YLL caused by higher temperatures from the 75th to the 99th percentile of the overall temperature range. The reference temperature was 19.7° C (75th percentile).
